# Editorial: Gut microbiome in black soldier fly (*Hermetia illucens* L.) larvae: symbiosis, function, and application

**DOI:** 10.3389/fmicb.2024.1441577

**Published:** 2024-06-21

**Authors:** Jibin Zhang, Sen Yang, Kashif ur Rehman

**Affiliations:** ^1^National Key Laboratory of Agricultural Microbiology, National Engineering Research Center of Microbial Pesticides, College of Life Science and Technology, Huazhong Agricultural University, Wuhan, China; ^2^Henan Agricultural University, Zhengzhou, China; ^3^German Institute of Food Technologies (DIL e.V.), Quakenbrück, Germany; ^4^Department of Microbiology, Faculty of Veterinary and Animal Sciences, The Islamia University of Bahawalpur, Bahawalpur, Pakistan

**Keywords:** protein metabolism, cellulose breakdown, microbiome engineering, nitrogen cycling, substrate influence on microbiota

The black soldier fly (*Hermetia illucens* L.) has attracted much interest due to its capacity to grow in habitats abundant with organic waste. The insect's ability to feed on decaying matter and its high tolerance for microorganisms make it an excellent choice for bioconversion operations. These procedures include converting organic waste into useful products, such as protein, fat, chitin, and bio-fertilizer. The symbiotic link between black soldier fly larvae (BSFL) and their gut microbiota plays a critical role in waste management, nitrogen cycling, and sustainable agriculture. The Research Topic titled “*Gut microbiome in black soldier fly (Hermetia illucens L.) larvae: symbiosis, function, and application*” presents innovative studies that shed light on the essential functions of gut microbiota in the bioconversion processes, metabolic functions, and overall ecological services offered by BSFL, and this editorial explores the functions of the gut microbiome of the BSFL, emphasizing the most recent research discoveries and their wider implications.

The collective findings of the publications emphasize the crucial function of BSFL gut microbiota in improving protein metabolism, cellulose breakdown, and overall bioconversion efficiency. The symbiotic association between BSFL and their intestinal microorganisms is vital for nutritional metabolism, namely in the breakdown and assimilation of proteins. The first article of this topic (Yu et al.) examined germ-free and gnotobiotic BSFL and found that the presence of certain gut microorganisms greatly enhances protein digestion and absorption. The protein reduction rate in gnotobiotic BSFL was 73.44% greater compared to their germ-free counterparts, providing evidence for the significance of microbial symbiosis in nutrition metabolism. The research found several gut bacteria, such as *Pseudomonas* spp., *Orbus* spp., *Campylobacter* spp., and *Dysgonomonas* spp., that had a substantial correlation with different elements of protein metabolism and enzyme function. *Dysgonomonas* spp. were specifically associated with the process of protein digestion and absorption, while *Issatchenkia* spp. had a significant association with pepsin activity. In addition, *Campylobacter* spp., *Pediococcus* spp., and *Lactobacillus* spp. were linked to trypsin activity, whereas *Lactobacillus* spp. and *Bacillus* spp. were connected with peptidase activity (Yu et al.).

Another research by Kariuki et al. observed the enzymatic functions occurring in the gut of BSFL when they were given diets high in lignocellulose. This work investigates the previously unexamined beneficial impacts of BSFL gut microbiomes and enzymes, especially carbohydrate-active enzyme (CAZyme) families, on the breakdown of lignocellulose. The findings revealed that BSFL raised on brewer's spent grain and wheat husk diets exhibited the greatest prevalence of *Bacteroides* and *Dysgonomonas*. The enzyme families GH51 and GH43_16, which consist of both α-L-*arabinofuranosidases* and exo- α-L-*arabinofuranosidase* 2, were often found in the digestive systems of BSFL that were fed with lignocellulosic diets. The presence of gene clusters producing hemicellulolytic *arabinofuranosidases* in the CAZy family GH51 was also detected, emphasizing their function in the degradation of intricate plant components. This highlights the capacity of BSFL to convert lignocellulosic waste into sugars, thereby enabling the production of valuable products (Kariuki et al.).

The comparative analysis presented by Auger et al. of the microbial communities in the gut of BSFL at various stages of development and on different substrates used for rearing. This research investigates the taxonomic makeup and changes in the microbiota associated with *H. illucens* throughout its life cycle, from eggs to second-generation adults. The study revealed that the kind of substrate (plant-based or animal-based) had a substantial impact on the makeup of the microbiota. The research found that the kind of substrate used for raising is the main factor influencing the makeup of microbiota resulted in unique profiles of bacteria and microeukaryotes, while the developmental stage influenced only whole individual's bacterial microbiota composition (Auger et al.). This study enhances our comprehension of the microbiota in *H. illucens* and provides a basis for optimizing microbiological conditions for industrial breeding. Moreover, the adaptability of BSFL gut microbiota to various industrial residual streams was examined by Vandeweyer et al., revealing the plasticity of these microbial communities. The findings showed a clear shift in bacterial community composition along the digestive tract, with the hindgut serving as a potential reservoir for core microbiota. This adaptability not only supports efficient digestion of diverse substrates but also highlights the potential for microbiome engineering to enhance BSFL performance on (industrial) residual streams (Vandeweyer et al.).

Finally, Shao et al. examined the process of converting Wuzhishan pig manure using BSFL and highlighted the influence of gut microbiota on the effectiveness of conversion. The inclusion of cellulose-degrading bacteria, such as *Bacillus cereus* and *Bacillus subtilis* greatly improved the breakdown of organic matter and increased BSFL bioconversion. The conversion of manure using BSFL, the changes in gut microbiota over time, and the potential benefits of thermophilic bacteria that break down cellulose. The results have significant ramifications for the sustainable management of waste and the effective conversion of biomass, which in turn contribute to the preservation of the environment and the recovery of resources (Shao et al.).

To summarize, the research presented in this Research Topic provides deep insights into the functional symbiosis between BSFL and their gut microbiota. These investigations significantly enhance our comprehension of how microbial populations influence nutrient metabolism and waste bioconversion in BSFL, offering new possibilities for sustainable waste management and resource recovery. As we further investigate and use the capabilities of these complex microbial ecosystems, the use of BSFL in environmental conservation and industrial bioprocessing is expected to grow, making a substantial contribution to global sustainability initiatives.

## Author contributions

JZ: Conceptualization, Writing – review & editing. SY: Writing – review & editing. KR: Writing – original draft, Writing – review & editing.

